# Simplified endoscopic septotomy for a large symptomatic Zenkerʼs diverticulum

**DOI:** 10.1055/a-2174-8690

**Published:** 2023-10-12

**Authors:** Ravishankar Asokkumar, Jeanette Pei Xuan Ng, Francisco Carlos Paolo Dimatatac, Yi Yuan Tan, Christopher Khor, Mark Cheah Chang Chuen

**Affiliations:** 1 Department of Gastroenterology and Hepatology, Singapore General Hospital, Singapore; 2 Duke-NUS Graduate Medical School, Singapore

Zenker’s diverticulum is a pulsion diverticulum occurring because of abnormal cricopharyngeal muscle (CPM) relaxation during swallowing ( Fig. 1 ) 1 . Traditionally, Zenker’s diverticulum has been treated by surgery; however, open surgery has high complication rates and morbidity. Flexible endoscopic options are a safe and effective treatment alternative for Zenker’s diverticulum, with comparable efficacy and low recurrence rates 2 . The Zenker’s diverticulum peroral endoscopic myotomy (zPOEM) and modified Z-POEM have been described as effective treatment options 3 . These procedures are technically demanding and require high levels of expertise. We present a simplified technique that was used to treat a large symptomatic Zenker’s diverticulum ( Video 1 ).

**Fig. 1:**
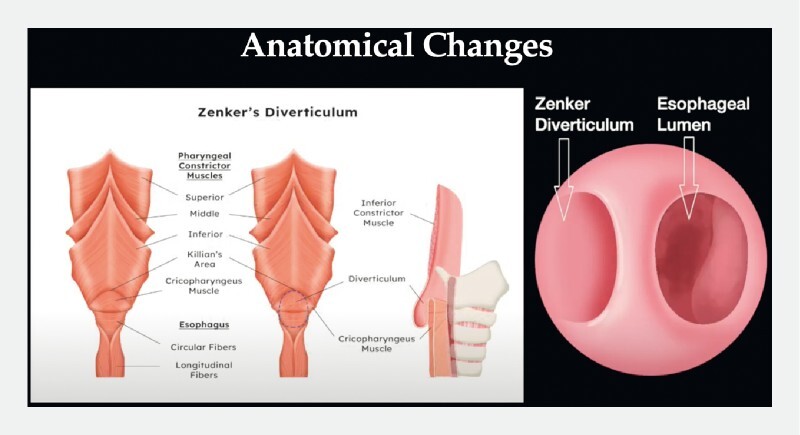
Illustration showing the anatomy of a Zenker’s diverticulum. Source: Lydia Tan Xin Rui.

**Video 1** Simplified endoscopic septotomy for a large symptomatic Zenker’s diverticulum. Source for graphical illustration: Lydia Tan Xin Rui.


A 79-year-old man presented with a 2-year history of dysphagia, weight loss, and regurgitation. Esophagogastroduodenoscopy showed a large (5 cm) Zenker’s diverticulum ( Fig. 2 ). A computed tomography scan and barium studies confirmed the large Zenker’s diverticulum, which was compressing the esophagus and occluding the lumen ( Fig. 3 ). The patient was therefore planned to undergo a simplified endoscopic septotomy under general anesthesia.

**Fig. 2:**
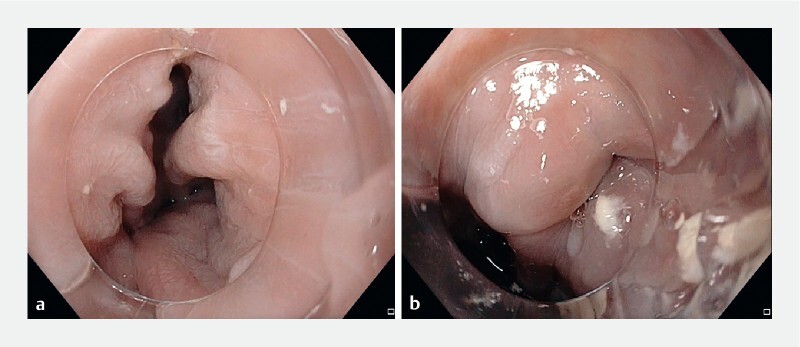
Endoscopic images showing: **a** a large Zenker’s diverticulum; **b** the septum and the compressed esophageal lumen.

**Fig. 3:**
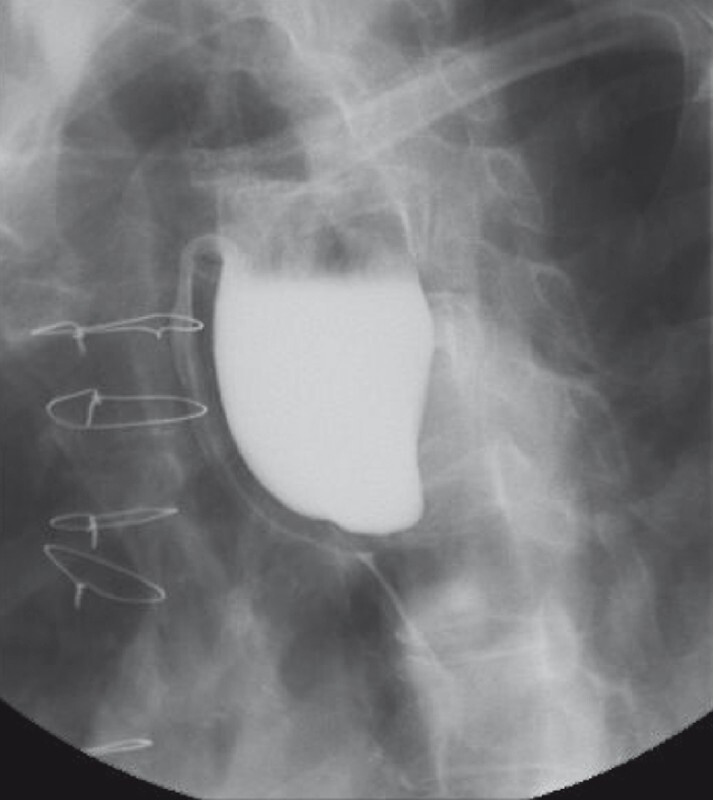
Barium swallow showing a large Zenker’s diverticulum causing compression of the esophagus.

We equipped the endoscope with a distal cap and used CO _2_ insufflation. First, we marked the esophageal lumen by advancing a feeding tube into it. Second, we cleaned the diverticular sac until it was free of debris. Third, we used a rotatable short insulated scissor-type knife (SB knife; Sumitomo Bakelite, Japan) to cut the septum and the CPM, using blended current for dissection. We started at the center and cut the septal mucosa and CPM sequentially to prevent flap formation. We regularly inspected the Zenker’s diverticulum and the esophageal lumen to ascertain the dissection depth, and ensured that the septum was dissected adequately to create a common space between the sac and the esophagus. Lastly, we secured hemostasis and closed the defect with clips ( Fig. 4 ). The patient was kept on a liquid diet and was discharged after 24 hours with analgesics.

**Fig. 4:**
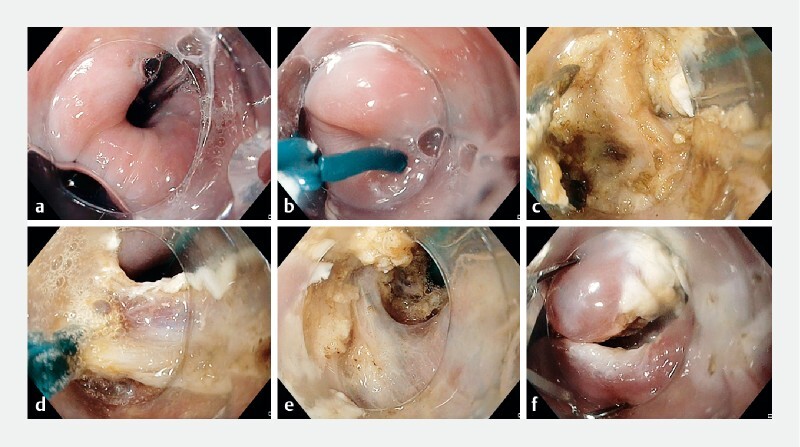
Endoscopic images during the simplified Zenker’s diverticulum peroral endoscopic myotomy (zPOEM) procedure showing: **a** the esophageal lumen marked with a feeding tube; **b** a scissor-type knife being used to cut the septum; **c** dissection of the mucosa to expose the cricopharyngeal muscle (CPM); **d** cutting of the CPM muscle; **e** dissection and cutting of the mucosa and the muscle until the base of the diverticulum is reached; **f** inspection of the base of the diverticulum, with hemostasis secured and the defect closed with clips.

In conclusion, our simplified stepwise approach is less technically challenging and could be quickly adopted to treat large Zenker’s diverticula safely.

Endoscopy_UCTN_Code_TTT_1AO_2AG

## Notes

Endoscopy E-Videos

https://eref.thieme.de/e-videos
*E-Videos* is an open access online section of the journal *Endoscopy* , reporting on interesting cases and new techniques in gastroenterological endoscopy. All papers include a high-quality video and are published with a Creative Commons CC-BY license. Endoscopy E-Videos qualify for HINARI discounts and waivers and eligibility is automatically checked during the submission process. We grant 100% waivers to articles whose corresponding authors are based in Group A countries and 50% waivers to those who are based in Group B countries as classified by Research4Life (see: https://www.research4life.org/access/eligibility/ ). This section has its own submission website at https://mc.manuscriptcentral.com/e-videos
